# Efficacy and safety of rasagiline in Chinese patients with early Parkinson’s disease: a randomized, double-blind, parallel, placebo-controlled, fixed-dose study

**DOI:** 10.1186/s40035-018-0137-5

**Published:** 2018-12-06

**Authors:** Zhenxin Zhang, Jian Wang, Shengdi Chen, Chunfeng Liu, Baorong Zhang, Rong Peng, Shenggang Sun, Xiangru Sun, Gang Zhao, Qiumin Qu, Yansheng Li, Suiqiang Zhu, Xiaoping Pan, Ming Shao, Yanping Wang

**Affiliations:** 1 Peking Union Medical College Hospital, Beijing, China; 2 Huashan Hospital, Fudan University, Shanghai, China; 3 Ruijin Hospital, Shanghai Jiaotong University School of Medicine, Shanghai, China; 4 The Second Affiliated Hospital of Soochow University, Suzhou, China; 5 The Second Affiliated Hospital, School of Medicine, Zhejiang University, Zhejiang, Hangzhou China; 6 West China Hospital, Sichuan University, Chengdu, China; 7 Tongji Hospital Affiliated to Tongji Medical College of Huazhong University of Science and Technology, Wuhan, China; 8 Peking University First Hospital, Beijing, China; 9 Xijing Hospital, The First Affiliated Hospital of The Fourth Military Medical University, Xi’an, China; 10 The First Affiliated Hospital of Xi’an Jiaotong University, Xi’an, China; 11 Renji Hospital, Shanghai Jiaotong University School of Medicine, Shanghai, China; 12 Tongji Hospital of Tongji Medical College, Huazhong University of Science and Technology, Wuhan, China; 13 Guangzhou First People’s Hospital, School of Medicine, South China University of Technology, Guangzhou, China; 14 The First Affiliated Hospital of Guangzhou Medical University, Guangzhou, China; 15 The Second Affiliated Hospital of Guangzhou Medical University, Guangzhou, China

**Keywords:** Parkinson’s disease, Monoamine oxidase inhibitor, Rasagiline, Monotherapy, China

## Abstract

**Background:**

Rasagiline is a monoamine oxidase-B inhibitor used for Parkinson’s disease (PD) treatment, but its effectiveness on Chinese patients is unclear. This study aimed to evaluate the efficacy and safety of rasagiline monotherapy in Chinese patients with early PD.

**Methods:**

A 26-weeks, randomized, double-blind, placebo-controlled study has been performed at 15 sites in China and enrolled outpatients (≥35 years old) with idiopathic PD without a history of using any dopaminergic drugs. Participants were randomized 1:1 to receive rasagiline 1 mg once daily or placebo. The primary endpoint was the change of the Unified Parkinson’s Disease Rating Scale (UPDRS) total score from baseline to 26 weeks treatment. Secondary endpoints included changes in UPDRS subscale scores from part I to III. Health status was assessed with the PD Questionnaire (PDQ)-39 and EuroQol-Five-Dimension (EQ-5D) questionnaire. Safety profile was collected until 30 weeks after randomization.

**Results:**

A total of 130 patients (*n* = 65/group) were recruited, and 127 (rasagiline, *n* = 64; placebo, *n* = 63) were included in the full analysis set. Baseline characteristics were comparable between the two groups. The decrease in the mean UPDRS total score was greater in the rasagiline group than in the placebo group (− 3.18 ± 0.95 vs. − 0.18 ± 0.98, *P* = 0.025), and the mean UPDRS part I non-motor symptoms score (− 0.54 ± 0.15 vs. -0.08 ± 0.15, *P* = 0.003) were significantly decreased in the rasagiline group compared with placebo treated patients. An improvement trend was observed in the active treatment group for the subscales evaluation with parts II and III, while the difference to placebo was not statistically significant. Life quality assessed by the EQ-5D visual analog scale improved in the rasagiline group but worsened in placebo treated patients. The overall incidence of treatment-emergent adverse events (AEs) was slightly lower in the rasagiline group (41.5%) than in the placebo group (46.2%).

**Conclusions:**

Rasagiline is effective, safe, and well tolerated as monotherapy for the treatment of Chinese PD patients.

**Trial registration:**

Clinicaltrials.gov: NCT01556165. Registered 13 Mar 2012.

**Electronic supplementary material:**

The online version of this article (10.1186/s40035-018-0137-5) contains supplementary material, which is available to authorized users.

## Background

Parkinson’s disease (PD) is a progressive neurodegenerative disorder caused by the loss of dopaminergic neurons of the substantia nigra pars compacta, which produces dopaminergic innervations to the striatum [1]. The cardinal features of PD include bradykinesia, muscular rigidity, resting tremor, and postural instability, which lead to disturbances of gait and falls [2]. PD prevalence in China is estimated at 1.7% in individuals aged 65 years or older [3]. A more recent meta-analysis estimated the overall prevalence and annual incidence in China to be 16–440 per 100,000 and 1.5–8.7 per 100,000 people, respectively [4].

Therapeutic agents for the motor symptoms of PD include levodopa (a precursor in dopamine synthesis), dopamine agonists and inhibitors of monoamine oxidase B (MAO-B), the major enzyme responsible for the oxidative metabolism of dopamine in the human brain. MAO-B inhibitors slow the loss of endogenous dopamine as monotherapy and also reduce the elimination of dopamine produced from levodopa when used as an adjunct therapy with levodopa [5]. Selegiline (or L-deprenyl) [6] is a first generation MAO-B inhibitor currently available in China. It is associated with high incidence of sleep disorder and taking it with a tyramine diet may increase hypertensive reactions [7, 8].

Rasagiline [N-propargyl-1(R)-aminoidan] is a potent, highly selective, and irreversible inhibitor of MAO-B, and shows good effectiveness for the treatment of PD in the Caucasian population. The clinical efficacy, safety and tolerability of rasagiline administration as monotherapy or adjunct therapy to levodopa have been established in several clinical studies. The TEMPO [9, 10] and ADAGIO [11] studies were randomized, double-blind, placebo-controlled, phase III trials of rasagiline as monotherapy for early PD; both studies demonstrated that rasagiline treatment is associated with lower total Unified Parkinson’s Disease Rating Scale (UPDRS) score [10], less functional decline [9] and reduced deterioration in the change of UPDRS score [11]. Moreover, a post-hoc analysis demonstrated that treatment with 1 mg/day rasagiline maintains motor function at baseline levels for over a year [12]. Nevertheless, current available study data fully support the improvement of rasagiline on motor symptoms of PD, there is still no clear evidence to indicate the effect of rasagiline on slowing the progression of PD [13].

No studies have specifically examined and manifested the efficacy, tolerability and safety of rasagiline monotherapy in Chinese patients with PD, although the efficacy of rasagiline administration as adjunct therapy to levodopa in this population has been confirmed [14, 15]. Therefore, this randomized, double-blind, parallel, placebo-controlled, fixed-dose study aimed to evaluate the efficacy and safety of rasagiline in Chinese patients with early PD not treated with levodopa.

## Materials and methods

### Study design and participants

This randomized, double-blind, parallel, placebo-controlled, fixed-dose clinical trial enrolled participants at 15 sites in China (Additional file 1) between March 2012 and December 2013. Subjects were Chinese outpatients with primary diagnosis of idiopathic PD according to established criteria [16], with at least two cardinal signs of PD without additional known or suspected causes of parkinsonism. All subjects were more than 35 years of age and had a Modified Hoehn and Yahr Staging Scale (HYSS) score less than 3 at screening and baseline. Subjects whose clinical condition at the time of enrollment allows them to stay for 26 weeks on placebo treatment according to the investigator’s judgment. The patients had no history or need of anti-Parkinson medications other than anticholinergics. The subjects who have not taken levodopa, dopamine agonists, or amantadine for at least 42 days before enrollment were considered to be recruited into this study.

All procedures were performed with the understanding and written consent of the subjects and with the approval of the institutional review boards at the participating institutes. This trial has been registered in clinicaltrials.gov as NCT01556165.

### Study intervention

After a 28-day screening period, the participants were randomized 1:1 to receive 1 mg of rasagiline or placebo once daily for 26 weeks, with a 4-week safety follow-up period. The patients were assigned treatment using a computer-generated randomized allocation schedule (implemented using an interactive voice/web response system; Almac Clinical Technologies, Craigavon, UK) with block randomization (block size of 4). The investigators, site staff and participants were blinded to medication assignment throughout the trial.

### Efficacy evaluation

Participants were examined at screening, at baseline, and at 4, 8, 14, 20 and 26 weeks after randomization. At each visit, an experienced and qualified independent investigator assessed patients disease severity using the UPDRS scale, including UPDRS mental, behavior and mood (part I), UPDRS activities of daily living (part II) and UPDRS motor examination (part III) scores [17]. Clinical Global Impressions-Severity (CGI-S) and CGI-Improvement (CGI-I) scales [18] were also used to evaluate disease severity and changes from baseline. Patients who required levodopa or treated with dopamine agonists were withdrawn. The participant’s health status was evaluated using Parkinson’s Disease Questionnaire (PDQ)-39 [19] and the EuroQol-Five-Dimension (EQ-5D) questionnaire [20], administered at baseline and 26 weeks, respectively.

The change of UPDRS total score from baseline to the 26 weeks was the primary endpoint. UPDRS parts I, II, and III subscales were used respectively to assess the changes of patients’ mental symptoms, activities of daily living, and motor symptoms from baseline to the endpoint. The responder was defined as the patient with a UPDRS total score reduced by > 3 points from baseline to week 26 [9, 10, 21, 22]. Changes of UPDRS subscales, CGI-S, and CGI-I score from baseline to each visit were recorded.

### Safety assessment

The participants were followed up until 4 weeks after the completion of the 26-week double-blind treatment. Vital signs and adverse events were monitored throughout the study. Safety assessments were based mainly on the occurrence, frequency, and severity of adverse events (AEs), and also included comprehensive measures such as vital signs, weight, electrocardiographic (ECG), physical examination, neurologic examination, and laboratory parameters. Laboratory assays were performed at Quintiles Medical Research and Development (Beijing) Ltd. (Beijing, China).

### Statistical analysis

Data were summarized by treatment group using descriptive techniques. Continuous variables were presented as mean ± standard deviation/standard error of the mean or median (minimum and maximum). Categorical variables were presented as count and percentage.

The primary endpoint was analyzed using an analysis of covariance (ANCOVA) model with treatment and center as fixed factors and baseline UPDRS total score as a covariate. The last observation carried forward (LOCF) method was used to handle missing data. A positive trend is shown if the estimated treatment difference is in favor of rasagiline for a two-sided test at the 25% level of significance. The estimated treatment difference was presented with 95% confidence interval (CI). The efficacy analysis included those patients with baseline data and at least one efficacy evaluation.

Changes from baseline in UPDRS total scores at each visit were analyzed using a mixed-effect models for repeated measures as in the primary efficacy analysis. The remaining UPDRS, CGI-S and CGI-I endpoints were analyzed using the primary endpoint model. For health status analysis, changes in EQ-5D and PDQ-39 scores were analyzed by ANCOVA by the LOCF method. *P* < 0.05 was considered statistically significant.

Safety was assessed in all randomized patients who received at least one dose of rasagiline by determining the number of AEs, serious AEs (SAEs), treatment-emergent AEs (TEAEs), AEs leading to withdrawal, and deaths in each treatment group.

The sample size was calculated to show significance in the primary efficacy analysis on a two-sided significance level of 0.25. Assuming a difference between rasagiline and placebo was a 3-point change in the UPDRS total score and a standard deviation on the change from baseline was 7 points, a sample size of 60 patients per group will guarantee an 88% probability to show the trend. Therefore, 130 patients were considered sufficient to ensure 120 patients for the primary efficacy analysis.

SAS® Version 9.2 (SAS Institute, Cary, NC, USA) was used for all statistical analyses.

## Results

### Demographic and clinical characteristics of the study participants at baseline

**Fig. 1:**
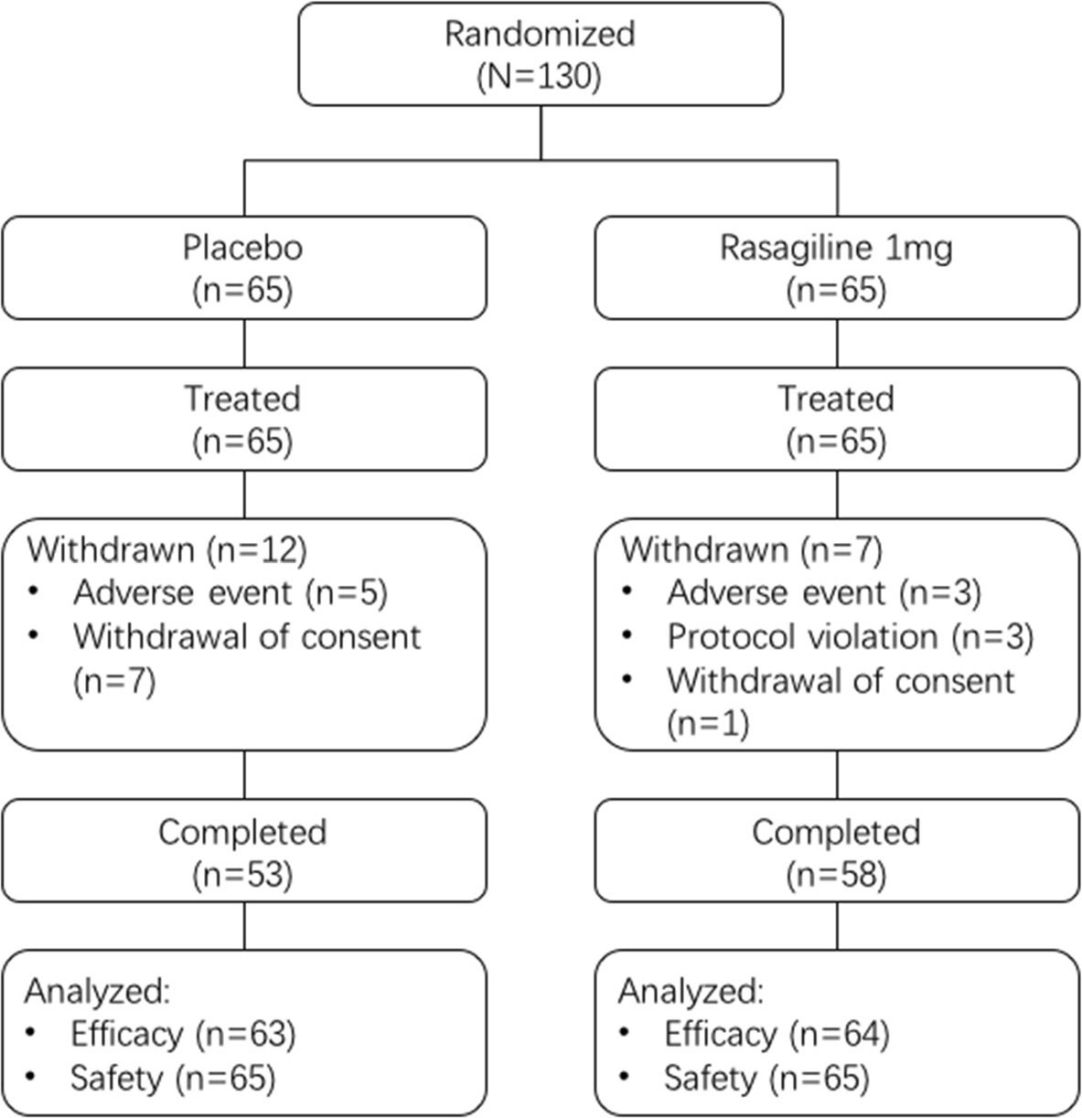
Flow diagram showing participant progression through various study phases

Of the 130 participants who were randomized to receive rasagiline 1 mg/d (*n* = 65) or placebo (*n* = 65), finally 127 (rasagiline group, *n* = 64; placebo group, *n* = 63) were included in the FAS analysis (Fig. 1). The demographic and clinical characteristics of patients at baseline included in the final analysis were comparable between the two groups (Table 1). All the patients were Chinese, and the mean age of the subjects was 59 years old. The mean Modified HYSS score was 1.6 ± 0.5 in both groups. No clinically relevant difference was observed in the baseline UPDRS total score between the rasagiline (26.14 ± 1.46) and placebo (28.56 ± 1.93) groups. Only one patient who taking levodopa during the study period was withdrawn due to adverse event.

**Table 1:** Demographic and clinical characteristics of the study participants at baseline

Characteristic	Placebo (*n* = 65)	Rasagiline 1 mg (*n* = 65)
Age (years)	59.5 ± 9.2	58.5 ± 8.7
Female	25 (38.5)	30 (46.2)
BMI (kg/m^2^)	22.7 ± 3.1	23.7 ± 2.8
Modified HYSS score	1.6 ± 0.5	1.6 ± 0.5
Disease duration (years)	0.11 (0.00, 5.46)	0.10 (0.00, 6.15)
UPDRS score		
Total	28.56 ± 1.93	26.14 ± 1.46
Part I	1.51 ± 0.20	1.58 ± 0.18
Part II	7.95 ± 0.58	7.24 ± 0.41
Part III	19.10 ± 10.60	17.33 ± 8.67
CGI-S score	3.1 ± 0.7	3.2 ± 0.6
MMSE total score	28.5 ± 1.6	28.8 ± 1.6
Patients with concurrent disorders	34 (52.3)	37 (56.9)

Data are mean ± standard deviation, *n* (%) or median (minimum, maximum). *BMI*, body mass index; *CGI-S*, Clinical Global Impressions Scale-Severity; *HYSS*, Hoehn and Yahr Staging Scale; *MMSE*, mini mental state examination; *UPDRS*, Unified Parkinson’s Disease Rating Scale

### Primary outcome

**Fig. 2:**
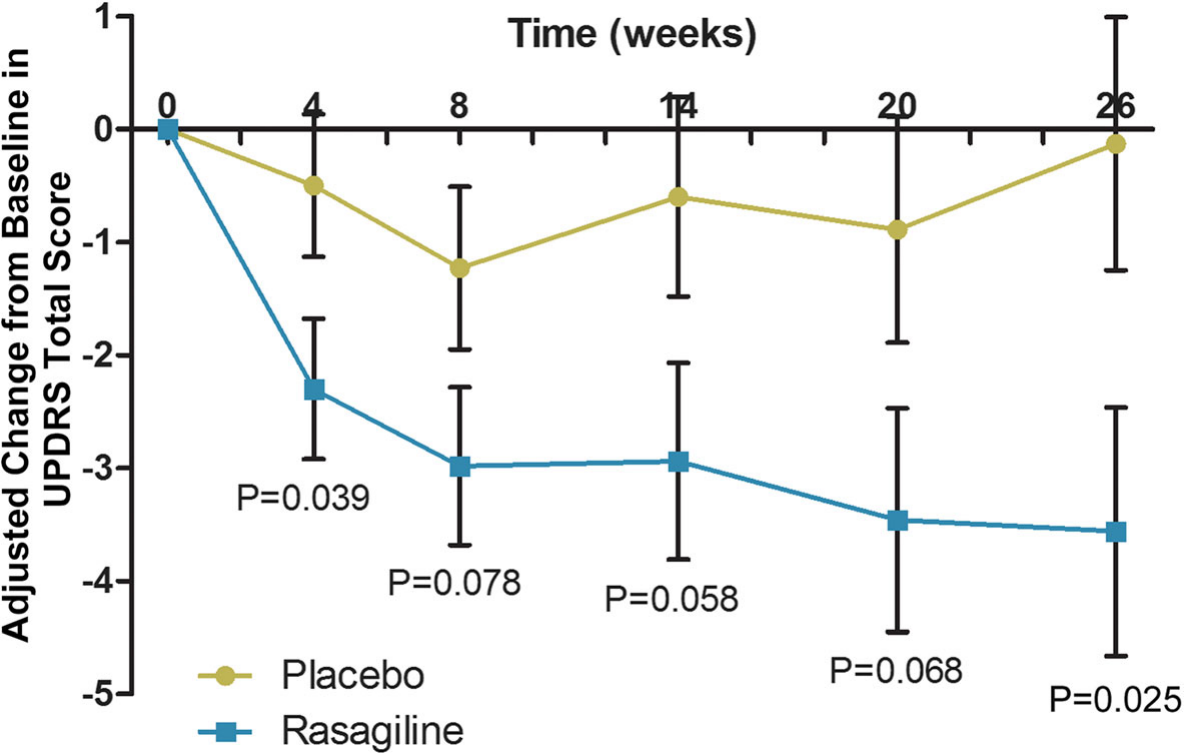
Adjusted Changes from baseline in Unified Parkinson’s Disease Rating Scale (UPDRS) total scores at each visit (full analysis set). Data are mean ± standard error

Patients with at least one post treatment UPDRS assessment were included in the primary efficacy full analysis set (FAS). After 26 weeks treatment, the adjusted mean changes from baseline in UPDRS total score were − 3.18 ± 0.95 points and − 0.18 ± 0.98 points in the rasagiline (*n* = 64) and placebo (n = 63) groups, respectively, and the treatment difference was statistically significant (*P* = 0.025; Table 2). Adjusted changes from baseline UPDRS total scores at each visit are shown in Fig. 2. The mean difference to placebo was statistically significant at weeks 4 and 26, and numerically in favor of rasagiline at the other time points but without statistical difference.

**Table 2:** Efficacy results at week 26 (FAS, LOCF)

Efficacy parameter	Change from baseline		Difference vs. placebo (95% CI)	*P* value
	Placebo (*n* = 63)	Rasagiline 1 mg (*n* = 64)		
Primary efficacy				
UPDRS total score	-0.18 ± 0.98	−3.18 ± 0.95	− 3.00 (− 5.62 to − 0.38)	0.025
Secondary efficacy				
UPDRS part I	0.08 ± 0.15	− 0.54 ± 0.15	−0.62 (− 1.03 to − 0.21)	0.003
UPDRS part II	0.25 ± 0.38	−0.43 ± 0.37	−0.67 (− 1.70 to 0.35)	0.196
UPDRS part III	−0.52 ± 0.68	− 2.23 ± 0.65	− 1.71 (− 3.52 to 0.10)	0.064
Exploratory efficacy				
Percent responders^a^	42 (66.7)	51 (79.7)		0.051
Odds ratio (95% CI)	2.55 (1.00 to 6.54)			
CGI-S score	0.08 ± 0.08	0.04 ± 0.08	−0.04 (− 0.25 to 0.17)	0.699
CGI-I score	3.88 ± 0.11	3.68 ± 0.11	− 0.20 (− 0.50 to 0.10)	0.196

Data are mean ± standard error of the mean or *n* (%).

^a^ Responders with worsening of < 3 points in *UPDRS* total score. *CGI-I*, Clinical Global Impressions Scale-Improvement; *CGI-S*, Clinical Global Impressions Scale-Severity; *CI,* confidence interval; *UPDRS*, Unified Parkinson’s Disease Rating Scale

### Secondary outcomes

The mean UPDRS part I score increased slightly in the placebo group and improved in the rasagiline group. The mean score increased from 1.51 ± 0.20 points to 1.63 ± 0.23 points in the placebo group, whereas the mean score decreased from 1.58 ± 0.18 points to 1.06 ± 0.16 points in the rasagiline group. The adjusted changes in UPDRS part I scores from baseline to week 26 were significantly different between the rasagiline (− 0.54 ± 0.15) and placebo (0.08 ± 0.15) groups (*P* = 0.003; Table 2). The mean difference to placebo at week 26 was numerically in favor of rasagiline for UPDRS-ADL and UPDRS motor subscales evaluation, but with no statistically significant difference (Table 2).

The proportion of responders was higher in the rasagiline group than in placebo treated patients although without statistically significant difference (79.7% vs. 66.7%, *P* = 0.051; Table 2). The overall status was assessed by the CGI-S and CGI-I scales, the mean of both assessments worsened slightly in the placebo group and the difference was in favor of rasagiline although without statistically significance (Table 2).

There were no differences between the two groups in PDQ-39 summary index and EQ-5D utility index scores, which deteriorated in both groups (Table 3). However, EQ-5D visual analog scale (VAS) scores, which reflect the participants’ perception of own health status, worsened in the placebo group but improved after rasagiline treatment (Table 3), indicating a statistically significant difference (*P* = 0.002).

**Table 3:** Participants’ health status at week 26 (FAS, ANCOVA, LOCF)

Health status	Placebo (*n* = 63)	Rasagiline 1 mg (*n* = 64)	*P* value
PDQ-39 Dimension			
Summary index	1.97 ± 1.15	0.77 ± 1.12	0.425
Activities of daily living			
Change from baseline	2.49 ± 1.82	1.86 ± 1.79	0.789
Bodily discomfort			
Change from baseline	1.28 ± 2.05	2.14 ± 2.01	0.749
Cognition			
Change from baseline	1.60 ± 1.91	−1.97 ± 1.87	0.156
Communication			
Change from baseline	2.10 ± 1.45	1.41 ± 1.42	0.716
Emotional well-being			
Change from baseline	1.43 ± 1.82	−1.62 ± 1.78	0.201
Mobility			
Change from baseline	3.13 ± 1.83	2.60 ± 1.79	0.823
Social support			
Change from baseline	2.91 ± 1.16	2.56 ± 1.14	0.819
Stigma			
Change from baseline	0.59 ± 2.16	−1.32 ± 2.12	0.502
EQ-5D score			
EQ-5D Utility index score			
Change from baseline	−0.04 ± 0.02	−0.01 ± 0.02	0.261
EQ-5D VAS score			
Change from baseline	−4.31 ± 1.65	2.49 ± 1.61	0.002

Data are mean ± standard error of the mean. *EQ-5D*, EuroQol-Five Dimension; *PDQ-39*, Parkinson’s Disease Questionnaire-39; *VAS*, visual analog scale

### Safety

The incidences of TEAEs and SAEs leading to withdrawal were slightly lower in the rasagiline group than in placebo treated individuals (Table 4). SAEs occurred only in the placebo group and were considered unrelated to the investigational product (4 participants had 5 SAEs, including cataract, major depression, road traffic accident, spinal osteoarthritis and thoracic vertebral fracture). TEAEs leading to withdrawal occurred in 5 patients (7.7%) of the placebo group and 3 (4.6%) in the rasagiline group. There were no deaths in either group. AE System Organ Classes with higher incidence rate in treatment arm were nervous system disorders (placebo: 15.4%; rasagiline: 20.0%) and eye disorders (placebo: 1.5%; rasagiline: 3.1%).

**Table 4:** Treatment emergent adverse effects in the study participants

Characteristic	Placebo (*n* = 65)	Rasagiline 1 mg (*n* = 65)
Patients with TEAEs	30 (46.2)	27 (41.5)
SAEs	4 (6.2)	0 (0)
TEAEs leading to withdrawal	5 (7.7)	3 (4.6)
Nervous system disorder	10 (15.4)	13 (20.0)
Bradykinesia	0 (0)	1 (1.5)
Dizziness	3 (4.6)	3 (4.6)
Headache	0 (0)	2 (3.1)
Parkinson’s disease^a^	4 (6.2)	5 (7.7)
Poor quality sleep	0 (0)	1 (1.5)
Somnolence	0 (0)	2 (3.1)
Speech disorder	1 (1.5)	0 (0)
Tremor	3 (4.6)	3 (4.6)
Gastrointestinal disorders	7 (10.8)	5 (7.7)
Abdominal discomfort	2 (3.1)	0 (0)
Constipation	0 (0)	1 (1.5)
Diarrhea	1 (1.5)	2 (3.1)
Dry mouth	1 (1.5)	1 (1.5)
Gastric dilation	1 (1.5)	0 (0)
Nausea	1 (1.5)	0 (0)
Psychiatric disorders	2 (3.1)	2 (3.1)
Hallucination	0 (0)	1 (1.5)
Insomnia	1 (1.5)	1 (1.5)
Major depression	1 (1.5)	0 (0)
Eye disorders	1 (1.5)	2 (3.1)

Data are *n* (%). *TEAE*, treatment-emergent adverse event. *SAE*, serious adverse event

^a^Parkinson’s disease means the worsening or progression of Parkinson’s disease

In both treatment groups, all mean laboratory test values were within the respective reference ranges at all time-points, and mean changes from baseline were negligible and not clinically relevant. Importantly, there were no major differences between the two groups.

## Discussion

The participants in this study were relatively young (mean age 59 years) with slight impairment in motor function, mild disease severity, and good cognitive function. In addition, all participants presented signs and symptoms of PD including tremor, rigidity, and bradykinesia and less than 10% exhibited postural disturbances. These characteristics are comparable with the symptoms observed in early PD patients.

The clinical efficacy, safety, and tolerability of rasagiline have been demonstrated in monotherapy studies of PD patients without treated by any anti-Parkinson medications [9–11]. This is the first study to investigate the efficacy and safety of rasagiline in early Chinese PD patients.

In the primary efficacy analysis, the reduction in mean UPDRS total score was significantly greater in the rasagiline group (3.2 points) than in the placebo controls (0.2 points). This difference of 3 points was slightly lower than 4.2 points observed after 26 weeks monotherapy with 1 mg/day rasagiline in Caucasian patients in the TEMPO study [10] but comparable to that reported by the ADAGIO study of Caucasian people (3.01 point difference) but treated with 1 mg/d rasagiline for a longer study period of 36 weeks [11]. Notably, the TEMPO study enrolled patients whose disease severity was Hoehn and Yahr stage 3 or lower, whereas the ADAGIO study included subjects with stage 2.5 or lower, same as in the current study. Although this study was limited to 26 weeks, a post-hoc analysis of the ADAGIO trial determined that the beneficial effects of rasagiline on UPDRS total score are maintained at 36 and 72 weeks, respectively, with motor function maintained at baseline levels for over a year [12, 23]. Furthermore, a follow-up study to the TEMPO trial found that 46% of patients could remain on rasagiline monotherapy for 2 years, i.e. without addition of other dopaminergic agents, with only 25% of patients progressing to Hoehn and Yahr stage 3 at 5.4 years [22]. Thus, the above findings in Chinese patients with early PD are consistent with those reported for Caucasian patients.

Next, the present study revealed that mental function assessed by the UPDRS I subscale, but not daily activities or motor symptoms assessed by the UPDRS II and III subscales, showed a significant improvement in the rasagiline group at week 26 compared with placebo treated patients. However, it should be noted that numerical values for UPDRS II and UPDRS III were in favor of rasagiline. Furthermore, UPDRS III (motor subscale) showed significantly higher value in the rasagiline group compared with placebo controls, a difference close to reaching statistical significance (*P* = 0.064). The current data are thus not entirely consistent with previous reports in Caucasian populations. The ADAGIO trial found that compared with placebo, 1 mg/day of rasagiline for 36 weeks resulted in significant improvements in all three UPDRS subscale scores [23]. The TEMPO study also reported significant differences between the rasagiline (1 mg/day) and placebo groups in the motor and activities of daily living subscales of UPDRS, although no difference was found in the mental subscale in contrast to the above results [10]. The lack of significant effects on UPDRS III and UPDRS II in this trial may be due to the small sample size, with this study being underpowered to detect real effects. Furthermore, the UPDRS is a relatively insensitive measure in subjects with early disease and may not capture improvement in motor and activities of daily living especially those manifestations with slight impairment since the average HYSS scores were lower as around 1.6 [24]. Disease severity may have also contributed to the lack of effects on UPDRS III and UPDRS II in this study, since a previous meta-analysis concluded that rasagiline has a greater effect in patients with more severe PD, i.e. individuals showing baseline UPDRS scores ≥27 [21], whereas the rasagiline group in the present study had a mean baseline UPDRS score of 26.14 ± 1.46. Interestingly, inconsistencies with the assessment of UPDRS total score and Hoehn and Yahr stage were observed in this study with TEMPO and ADAGIO (present study: 26, 1.6; TEMPO: 25, 1.9; ADAGIO: 20, 1.5). This evaluation discrepancy may also contribute to the slight inconsistency for China study UPDRS subscale assessment with TEMPO and ADAGIO. However, our study and other previous studies consistently confirm the efficacy of rasagiline monotherapy in early PD patients.

The ratio of responders was also higher in the rasagiline group than in the placebo group (borderline significance), as was also reported in the TEMPO study [10]. This study found no significant effect of rasagiline on CGI-S and CGI-I. However, the PRESTO study found an improvement in CGI after treatment for 26 weeks with 1 mg/day rasagiline [25]. The reasons for the apparent discrepancy between this trial and the PRESTO study are unknown, although the subjective nature of the CGI (which results in inconsistency) [26] and the smaller sample size in this study may have been contributing factors by reducing the statistical power.

Rasagiline has been reported to improve non-motor symptoms [23, 27, 28] and the quality of life [29, 30] in patients with PD. In the present work, the participants’ self-rated health (EQ-5D VAS) was significantly improved in the rasagiline group compared with placebo treated patients. Although there were no significant differences in any of the PDQ-39 dimensions between the rasagiline and placebo groups, it was evident that the improvements observed after 26 weeks were only in the rasagiline group for cognition, emotional wellbeing and stigma. The TEMPO study observed an improvement of patient’s quality of life, as reflected by the PDQUALIF summary score, in individuals treated with rasagiline [10].

In this study, incidence rates of TEAEs were comparable between the rasagiline and placebo groups, and the majority of TEAEs were mild or moderate. The two TEAEs with an incidence ≥5% in both groups were accidental overdose and PD progression. With regard to tolerability, the proportion of withdrawals in the rasagiline group was low (11%), and there were few withdrawals due to AEs. In addition, there were no clinically relevant changes or differences over time between the two treatment groups in vital signs, weights or electrocardiographic findings.

This study had some limitations. First, the sample size was small, and the study may have been underpowered to detect the actual differences between the rasagiline and placebo groups. Secondly, the study lasted only 26 weeks. Therefore, long-term efficacy and safety of rasagiline in a bigger Chinese populations remain to be established.

## Conclusions

In conclusion, 1 mg/day of rasagiline monotherapy for 26 weeks is effective to improve the global health status of Chinese patients with early PD. Furthermore, rasagiline is safe and well tolerated.

## Additional file

Additional file 1: List of study sites. (DOC 32 kb)

## Abbreviations

AEs: Adverse events
CGI-I: CGI-Improvement
CGI-S: Clinical Global Impressions-Severity
CI: Confidence interval
ECG: Electrocardiographic
EQ-5D: EuroQol-Five-Dimension
FAS: Full analysis set
HYSS: Hoehn and Yahr Staging Scale
LOCF: Last observation carried forward
MAO-B: Monoamine oxidase B
PD: Parkinson’s disease
PDQ: PD Questionnaire
SAEs: Serious AEs
TEAEs: Treatment-emergent AEs
UPDRS: Unified Parkinson’s Disease Rating Scale
VAS: Visual analog scale

## References

[1] Gazewood JD, Richards DR, Clebak K (2013). Parkinson disease: an update. Am Fam Physician.

[2] Lee A, Gilbert RM (2016). Epidemiology of Parkinson disease. Neurol Clin.

[3] Zhang ZX, Roman GC, Hong Z, Wu CB, Qu QM, Huang JB, Zhou B, Geng ZP, Wu JX, Wen HB (2005). Parkinson's disease in China: prevalence in Beijing, Xian, and Shanghai. Lancet.

[4] Zou YM, Liu J, Tian ZY, Lu D, Zhou YY (2015). Systematic review of the prevalence and incidence of Parkinson's disease in the People's Republic of China. Neuropsychiatr Dis Treat.

[5] Dezsi L, Vecsei L (2017). Monoamine oxidase B inhibitors in Parkinson's disease. CNS & neurological disorders drug targets.

[6] Fabbrini G, Abbruzzese G, Marconi S, Zappia M (2012). Selegiline: a reappraisal of its role in Parkinson disease. Clin Neuropharmacol.

[7] Yasar S, Goldberg JP, Goldberg SR (1996). Are metabolites of l-deprenyl (selegiline) useful or harmful? Indications from preclinical research. J Neural Transm Suppl.

[8] Churchyard A, Mathias CJ, Boonkongchuen P, Lees AJ (1997). Autonomic effects of selegiline: possible cardiovascular toxicity in Parkinson's disease. J Neurol Neurosurg Psychiatry.

[9] Parkinson Study G (2004). A controlled, randomized, delayed-start study of rasagiline in early Parkinson disease. Arch Neurol.

[10] Parkinson Study G (2002). A controlled trial of rasagiline in early Parkinson disease: the TEMPO study. Arch Neurol.

[11] Olanow CW, Rascol O, Hauser R, Feigin PD, Jankovic J, Lang A, Langston W, Melamed E, Poewe W, Stocchi F (2009). A double-blind, delayed-start trial of rasagiline in Parkinson's disease. N Engl J Med.

[12] Jankovic J, Berkovich E, Eyal E, Tolosa E (2014). Symptomatic efficacy of rasagiline monotherapy in early Parkinson's disease: post-hoc analyses from the ADAGIO trial. Parkinsonism Relat Disord.

[13] Oertel WH (2017). Recent advances in treating Parkinson's disease. F1000Research.

[14] Zhang L, Zhang Z, Chen Y, Qin X, Zhou H, Zhang C, Sun H, Tang R, Zheng J, Yi L (2013). Efficacy and safety of rasagiline as an adjunct to levodopa treatment in Chinese patients with Parkinson's disease: a randomized, double-blind, parallel-controlled, multi-Centre trial. Int J Neuropsychopharmacol.

[15] Zhang Z, Shao M, Chen S, Liu C, Peng R, Li Y, Wang J, Zhu S, Qu Q, Zhang X (2018). Adjunct rasagiline to treat Parkinson's disease with motor fluctuations: a randomized, double-blind study in China. Translational Neurodegeneration.

[16] Hughes AJ, Daniel SE, Kilford L, Lees AJ (1992). Accuracy of clinical diagnosis of idiopathic Parkinson's disease: a clinico-pathological study of 100 cases. J Neurol Neurosurg Psychiatry.

[17] Fahn S, Elton R, Fahn S, Marsden C, Calne D, Goldstein M (1987). Members of the UPDRS Development Committee: The Unified Parkinson’s Disease Rating Scale. *Recent developments in Parkinson’s disease. Volume 2*.

[18] Busner J, Targum SD (2007). The clinical global impressions scale: applying a research tool in clinical practice. Psychiatry.

[19] Peto V, Jenkinson C, Fitzpatrick R (1998). PDQ-39: a review of the development, validation and application of a Parkinson's disease quality of life questionnaire and its associated measures. J Neurol.

[20] Rabin R, de Charro F (2001). EQ-5D: a measure of health status from the EuroQol group. Ann Med.

[21] Hauser RA, Abler V, Eyal E, Eliaz RE (2016). Efficacy of rasagiline in early Parkinson's disease: a meta-analysis of data from the TEMPO and ADAGIO studies. The International journal of neuroscience.

[22] Lew MF, Hauser RA, Hurtig HI, Ondo WG, Wojcieszek J, Goren T, Fitzer-Attas CJ (2010). Long-term efficacy of rasagiline in early Parkinson's disease. The International journal of neuroscience.

[23] Rascol O, Fitzer-Attas CJ, Hauser R, Jankovic J, Lang A, Langston JW, Melamed E, Poewe W, Stocchi F, Tolosa E (2011). A double-blind, delayed-start trial of rasagiline in Parkinson's disease (the ADAGIO study): prespecified and post-hoc analyses of the need for additional therapies, changes in UPDRS scores, and non-motor outcomes. The Lancet Neurology.

[24] Skorvanek M, Martinez-Martin P, Kovacs N, Rodriguez-Violante M, Corvol J, Taba P (2017). Differences in MDS-UPDRS scores based on Hoehn and Yahr stage and disease duration. Mov Disord Clin Pract.

[25] Parkinson Study G (2005). A randomized placebo-controlled trial of rasagiline in levodopa-treated patients with Parkinson disease and motor fluctuations: the PRESTO study. Arch Neurol.

[26] Forkmann T, Scherer A, Boecker M, Pawelzik M, Jostes R, Gauggel S (2011). The clinical global impression scale and the influence of patient or staff perspective on outcome. BMC psychiatry.

[27] Stocchi F (2014). Investigators a: benefits of treatment with rasagiline for fatigue symptoms in patients with early Parkinson's disease. Eur J Neurol.

[28] Panisset M, Stril JL, Belanger M, Lehoux G, Coffin D, Chouinard S (2016). Open-label study of sleep disturbances in patients with Parkinson's disease treated with Rasagiline. The Canadian journal of neurological sciences Le journal canadien des sciences neurologiques.

[29] Biglan KM, Schwid S, Eberly S, Blindauer K, Fahn S, Goren T, Kieburtz K, Oakes D, Plumb S, Siderowf A (2006). Rasagiline improves quality of life in patients with early Parkinson's disease. Movement disorders : official journal of the Movement Disorder Society.

[30] Jost WH, Klasser M, Reichmann H (2008). Rasagiline in daily clinical use. Results of a treatment study of Parkinson patients with a combination treatment. Fortschr Neurol Psychiatr.

