# Supplementary material for: Efficacy and safety of rasagiline in Chinese patients with early Parkinson’s disease: a randomized, double-blind, parallel, placebo-controlled, fixed-dose study
Source: Transl Neurodegener. 2018 Dec 6;7:32. doi: 10.1186/s40035-018-0137-5 (PMC6282325; doi:10.1186/s40035-018-0137-5)
Supplement: Supplementary file 1 — List of study sites. (DOC 32 kb) [file 40035_2018_137_MOESM1_ESM.doc]

**Additional file 1. List of study sites**

| Site No. | Hospital Name |
| --- | --- |
| CN001 | Peking Union Medical College Hospital |
| CN002 | First Affiliated Hospital, Chongqing Medical University |
| CN003 | First Hospital Affiliated to Guangzhou Medical University |
| CN004 | Second Affiliated Hospital of Zhejiang University College of Medicine |
| CN005 | Guangzhou First Municipal People’s Hospital |
| CN006 | Second Affiliated Hospital of Soochow University |
| CN007 | Huashan Hospital, Fudan University |
| CN009 | Wuhan Union Hospital, Tongji Medical College of Huazhong University of Science & Technology |
| CN010 | Xijing Hospital, First Affiliated Hospital of The Fourth Military Medical University |
| CN011 | West China Hospital of Sichuan University |
| CN012 | Ruijin Hospital Affiliated to Shanghai Jiaotong University School of Medicine |
| CN013 | Renji Hospital Affiliated to Shanghai Jiaotong University School of Medicine |
| CN014 | First Affiliated Hospital of Medical College of Xi'an Jiaotong University |
| CN015 | Peking University First Hospital |
| CN016 | Tongji Hospital, Tongji Medical College of Huazhong University of Science & Technology |
